# Genomic Characterization of CIAV Detected in Contaminated Attenuated NDV Vaccine: Epidemiological Evidence of Source and Vertical Transmission From SPF Chicken Embryos in China

**DOI:** 10.3389/fvets.2022.930887

**Published:** 2022-07-06

**Authors:** Yan Li, Jinjin Wang, Longfei Chen, Qun Wang, Meng Zhou, Hui Zhao, Zengna Chi, Yixin Wang, Shuang Chang, Peng Zhao

**Affiliations:** College of Veterinary Medicine, Shandong Agricultural University, Tai'an, China

**Keywords:** chicken infectious anemia virus, vertical transmission, SPF chicken, vaccine, genome analysis, molecular characterization

## Abstract

Live attenuated vaccines have been extensively used to prevent infectious disease in poultry flocks. Freedom from exogenous virus is a high priority for any veterinary vaccines. Recently, attenuated Newcastle disease virus (NDV) vaccines were detected to be contaminated with chicken infectious anemia virus (CIAV) in a routine screening for exogenous viruses. To investigate the possible source of the contamination, we conducted virological tests on a specific-pathogen-free (SPF) layer breeder flock that provide the raw materials for vaccines in this manufacturer. Firstly, CIAV antibodies in serum and egg yolks samples of the SPF laying hens were detected by ELISA assays. The results showed that CIAV antibodies in serum and egg yolks were 62% positive and 57% positive, respectively. Then, DNA was extracted from the NDV vaccines and SPF chicken embryonated eggs, and detected by molecular virology assays. The results showed that three assays for pathogens in embryonated eggs had similar positive rates (35.8%). And the sequences of CIAV from SPF embryos and NDV vaccines consisted of 2,298 nucleotides (nt) with 100% homology. The new full-length genome of CIAV was designated SDSPF2020 (Genbank accession number: MW660821). Data showed SDSPF2020 had the sequence similarities of 95.8–99.6% with reference strains, and shared the highest homology with the Chinese strain HLJ15125. These results strongly suggested that exogenous CIAV contamination is most likely caused by wild virus infection in SPF flocks and vertical transmission to chicken embryos. Collectively, this study illustrated that vertical transmission of CIAV from a SPF layer breeder flock to embryos was a non-neglible way for exogenous virus contamination in vaccine production.

## Introduction

Chicken infectious anemia virus (CIAV) can cause infectious anemia disease characterized by growth retardation and immunosuppression in chickens, which brings costly losses in the poultry industry (1, 2). In the 1970 s, CIAV was first isolated from contaminated vaccines in Japan (3), and its epidemiological and pathogenic features have been revealed as research continues (4–6). Previous studies have shown that CIAV can spread through multiple routes and induce immune dysfunction accompanied by severe damage of the hemocytoblast and T cell lineages (7–9). CIAV is highly prevalent in chicken flocks and infects chickens of all ages through horizontal transmission (10–12), but young chicks are more susceptible to CIAV infection and lesions due to the underdeveloped immune system (8, 13, 14). Infection of 2-week-old chickens resulted in classic symptoms of immunosuppressive disease such as aplastic anemia, heterophil decrease, lymphoid depletion and atrophy of bone marrow hematopoietic tissue (8). Cardona et al. reported that viral DNA hidden in the gonadal tissues of SPF breeding flocks could be transferred to newborn eggs without early viral replication (15). In addition, CIAV carried by feather shafts or within the pulp of feathers can be transmitted horizontally to chickens via the mucosal entries causing diseases (16). Even under good biosafety condition, maintaining CIAV seronegative poultry population is highly challenging due to extreme resistance of CIAV to physical and chemical treatments (17, 18). Hence, stricter monitoring and control systems should be established to prevent the infection and transmission of CIAV and reduce economic losses for poultry farming.

The epidemiological characteristics and pathogenicity of CIAV depends on its molecular structure (19, 20). CIAV is a non-enveloped virus with a single-stranded circular DNA genome containing three partially overlapping ORFs, including VP1-, VP2- and VP3-coding regions (5, 21, 22). VP1 (51.6 kDa) is a viral capsid protein related to the formation of neutralizing antibody, and its conformation is affected by VP2 (24.0 kDa), a non-structural scaffold protein (23, 24). VP3 (13.6 kDa), also known as apoptin, induces apoptosis of thymic precursors and hemocytoblasts (4). Notably, amino acids (AAs) of VP1 involved in the pathogenesis of CIAV are prone to variability, while VP2 and VP3 are relatively conserved. However, VP1 nucleotide or amino acid sequences show fine variation (<6%) between different strains separated by time and geographical location (25, 26). Besides, the non-coding region of CIAV genome contains a series of conserved motifs that exhibit promoter activity and play a crucial role in viral replication and transcription (1, 27–29). Thus, phylogenetic analysis of CIAV complete genome and VP1 sequences is a significant step to understand the evolutionary branches and reveal the differences of amino acids and transcription elements between different strains.

Recently, epidemiological investigation reported that CIAV has sporadically emerged in vaccinated and/or unvaccinated commercial chicken flocks in China (30–33), and the use of CIAV-contaminated attenuated vaccines was suspected to be an important route of transmission. Exogenous CIAV as contaminant in commercial attenuated vaccines has been documented in previous investigations (34, 35). However, no evidence was available to confirm the source of exogenous CIAV contamination in vaccines. To address this issue, we investigated the source of re-emerged CIAV contamination in NDV vaccines and conducted systemic experiments to find a complete chain of evidence for the transmission of CIAV from a SPF layer breeder flock to embryos, and eventually, to the live-attenuated NDV vaccines.

## Materials and Methods

### Background of CIAV Contamination in Vaccines

According to the requirements of Ministry of Agriculture and Rural Affairs of the People's Republic of China, all live poultry vaccines must be regularly tested for exogenous virus dissemination before marketing. To identify if the potential threat caused by live vaccines contaminated with any exogenous virus, we investigated commercial live poultry vaccines produced by a vaccine manufacturer with SPF chicken breeder flocks. Three batch of live vaccines against NDV were provided by the vaccine manufacturer, and three bottles were randomly sampled from each batch. SPF chicks were orally vaccinated with the NDV vaccines at 7 days of age, and antibody responses were detected at 6 wk post-inoculation using commercial enzyme-linked immunosorbent assay (ELISA) kits (Avian Leukosis Virus Antibody Test Kit-subgroup A/B, Avian Leukosis Virus Antibody Test Kit-subgroup J, Reticuloendotheliosis virus Antibody Test Kit, Chicken infectious anemia virus Antibody Test Kit, IDEXX, USA). SPF chicks were positive for CIAV antibody after inoculation with NDV vaccines. Subsequently, DNAs were extracted from NDV vaccines to amplify the CIAV genome using commercial TIANamp Genomic DNA Kits (Tiangen Biotechnology Co., Ltd., China). Each experiment was repeated at least three times. According to the published whole genome sequences of CIAVs in GenBank (Table 1), a pair of primers (Table 2) were designed and synthesized to detect the viral DNA.

**Table 1:** The sequence information of CIAV reference strains used in this work.

**No**.	**Isolate**	**Origin**	**Year**	**Accession no**.
1	01–4201	USA	2001	DQ991394
2	704	Australia	1996	U65414
3	17SY0902	China	2017	MK089243
4	26P4	USA	1991	D10068
5	3–1	Malaysia	2000	AF390038
6	98D02152	USA	1997-1998	AF311892
7	98D06073	USA	1997-1998	AF311900
8	AB1K	Turkey	2016	MT259319
9	AH4	China	2005	DQ124936
10	AH6	China	2005	DQ124935
11	AH-C369	Japan	2000	AB046590
12	BD-3	Bangladesh	2001	AF395114
13	BJ0401	China	2005	DQ124934
14	C14	China	2002	EF176599
15	C140	Japan	2000	AB046587
16	CAA82-2	Japan	1987	D31965
17	CIA-1	USA	1988	L14767
18	CIAV-10	Argentina	2007	KJ872513
19	CIAV-18	Argentina	2007	KJ872514
20	CIAV-4	China	2012	KJ728816
21	CIAV89-69	Korea	1991	JF507715
22	Cloned isolate 10	UK	1996	U66304
23	Cux-1	Germany	1990	M55918
24	Cuxhaven-1	Germany	1983	M81223
25	Del-Ros	USA	2000	AF313470
26	DI072479	USA	1990	DI072479
27	G6	Japan	2003	AB119448
28	GD-101	China	2014	KU050680
29	GD-104	China	2014	KU050679
30	GD-B-12	China	2011	KF224926
31	GD-K-12	China	2012	KF224935
32	GX1804	China	2018	MK484615
33	GX1904B	China	2019	MN103406
34	Harbin	China	2002	AF475908
35	HLJ15125	China	2015	KY486139
36	HN9	China	2005	DQ141672
37	Isolate 1	China	2012	KJ728814
38	Isolate 18	China	2012	KJ728827
39	Isolate 22	China	2012	KJ728830
40	Isolate 6	China	2012	KJ728817
41	LF4	China	2004	AY839944
42	LN15170	China	2015	KY486155
43	N8	China	2016	MK887164
44	SC-SM	China	2014	KM496305
45	SD1403	China	2014	KU221054
46	SD15	China	2015	KX811526
47	SD1509	China	2015	KU645510
48	SD22	China	2005	DQ141673
49	SD24	China	2005	AY999018
50	SDLY08	China	2008	FJ172347
51	SH11	China	2005	DQ141670
52	SH16	China	2005	DQ141671
53	SK4	Egypt	2017	MG827100
54	SMSC-1	Malaysia	2000	AF285882
55	SMSC-1P60	Malaysia	2001	AF390102
56	TJBD33	China	2004	AY843527
57	TJBD40	China	2004	AY846844
58	TR20	Japan	1999	AB027470
59	UT-Zahraee	Iran	2018	MT239353
60	WO9603507	USA	1996	A48606
61	SDSPF2020	China	2020	MW660821

**Table 2:** Sequences of primers used for amplification in this study.

**Primers**	**The sequences of the primers (5' → 3')**	**Location**	**Sizes**
CIAV-T-F^a^	5′- CGCTCTCCAAGAAGATACTC - 3′	470–1,150	682bp
CIAV-T-R	5′- CTGAAATCTTGGCGACTCTC - 3′		
CIAV-F^b^	5′- GCATTCCGAGTGGTTACTATTCC - 3′	1–942	942bp
CIAV-R	5′- TCTCCTCCGATGTCGAAATTTATA - 3′		
CIAV-F_D_^c^	5′- GTTACTATTCCATCACCATT - 3′	13–682	670bp
CIAV-R_D_	5′- ACATTCTTGAAACCAGTGCT - 3′		
C-F^d^	5′- GCATTCCGAGTGGTTACTATTCC - 3′	1–843	843bp
C-R	5′- CGTCTTGCCATCTTACAGTCTTAT - 3′		
CC-F^d^	5′- TACGTCACAGCCAATCAGAA - 3′	231–648	418bp
CC-R	5′- GCATTGCAGATCTTAGCGT - 3′		
CIAV-q-F^e^	5′- CGGATTGGTATCGCTGGA - 3′	564–718	155bp
CIAV-q-R	5′- GAGGGAGGCTTGGGTTGAT - 3′		
CIAV-com-F1^f^	5′- GCATTCCGAGTGGTTACTATTCC - 3′	1–842	842bp
CIAV-com-R1	5′- CGTCTTGCCATCTTACAGTCTTAT - 3′		
CIAV-com-F2^f^	5′- CGAGTACAGGGTAAGCGAGCTAAA−3′	743–1,732	990bp
CIAV-com-R2	5′- TGCTATTCATGCAGCGGACTT - 3′		
CIAV-com-F3^f^	5′- ACGAGCAACAGTACCCTGCTAT - 3′	1,643–150	802bp
CIAV-com-R3	5′- CTGTACATGCTCCACTCGTT - 3′		

*^a^Primers were used to identify CIAV gene sequence*.

*^b^Primers to generate PCR products for the dot blot hybridization assay*.

*^c^Primers used for Digoxigenin-DNA probe amplification*.

*^d^Outer and inner primers for nested-PCR*.

*^e^Primers for SYBR Green I-based real-time PCR assay*.

*^f^Three pairs of primers were designed to amplify three overlapping fragments (36)*.

### Detection of CIAV Antibody in SPF Chickens

To determine the source of CIAV contamination in live NDV vaccines, blood samples (*n* = 1,000) were collected from a SPF layer breeder flock. Serum was harvested using standard procedures and stored at 4°C until tested in an ELISA assay according to the manufacturer's instructions (Chicken Infectious Anemia Virus Antibody Test Kit, IDEXX, USA). Next, yolks of SPF chicken embryos (*n* = 200) used for vaccine production were randomly collected, and maternal CIAV antibody was detected with the dilution ratio of 1:20 using commercial ELISA kit (37).

### DNA Extraction and CIAV Detection in Chicken Embryos

Fourteen to 18 day old SPF chicken embryos (*n* = 40) of the same production origin were randomly selected, and samples of pooled organs (liver, spleen and thymus) were excised from each embryo. The tissues were homogenized in phosphate-buffered saline (PBS), and DNA was extracted using dna extraction kits (Tiangen, China) according to the manufacturer's instructions. The extracted DNA was stored in TE buffer (10 mM Tris, pH 7.5; 1 mM EDTA, pH 8.0) at −20°C. Based on the CIAV genome sequences published in Genbank, specific primers targeted to the conserved regions of viral genome were designed by using Primer 5.0 (Table 2) (38, 39), and used for PCR. PCR products amplified from viral DNA in the samples were detected by a dot blot hybridization assay. PCR products and controls were spotted onto the nitrocellulose membrane as dots, then baked at 80°C in a vacuum oven and hybridized with a Digoxigenin-labeled DNA probe generated by PCR (PCR-DIG Probe Synthesis Kit, Roche Diagnostics, USA) using primers CIAV-FD/RD (Table 2). Meanwhile, high-sensitivity nested-PCR and quantitative real-time polymerase chain reactions (qPCR) were parallelly performed for CIAV detection using the previous published methods (36, 40). The primers used are shown in Table 2. Three replicates of each sample were tested by each of the three different methods.

### Viral Genome Amplification and Sequencing

Three overlapping genomic segments were amplified from DNA extracted from the PCR-positive pooled organs using three pairs of primers (Table 2) (41) to establish the full-length nucleotide sequences. The PCR amplification was carried out in a 25μL total PCR reaction volume containing 10 pmol of each primer, 2.5 μL 10 × PCR buffer (Mg^2+^), 2 μL dNTPs (2.5 mM), 0.5 μL rTaq DNA polymerase (TaKaRa Biotechnology Co. Ltd., Dalian, China), under the following protocol: denaturation at 95°C for 5 min, followed by 34 cycles of 95°C for 30 s, 56°C for 45 s, and 72°C for 1 min, and a final elongation step at 72°C for 7 min. The PCR products were purified by agarose gel electrophoresis and subcloned into the pMD-18T vector (Takara-Bio, Dalian, China). Genomic DNA extracted from the attenuated NDV vaccines was amplified and sequenced simultaneously. Three genomes from different SPF chicken embryos were randomly selected to amplify, and three clones of each PCR product were randomly selected and submitted to Tsingke Biotechnology Co., Ltd. (Qingdao, China) for sequencing by Sanger method.

### Alignment and Phylogenetic Analysis of Viral Genome Sequence

The complete genome sequences of the new strain were obtained by assembling separated overlapping fragments using DNAstar software (version 7.1), and multiple sequence alignment was performed with the new strain and 60 reference genome sequences downloaded from GenBank (Table 1) using Clustal W method. Homology analysis was implemented by MegAlign and BLAST (https://blast.ncbi.nlm.nih.gov/Blast.cgi). The phylogenetic trees were generated by the neighbor-joining (NJ) method using MEGA X based on the full-length genome, VP1, VP2, and VP3 regions. Bootstrap values (1,000 replications) were indicated on each tree. And phylogenetic trees were further displayed, manipulated, and annotated with a web-based tool (Interactive Tree of Life http://itol.embl.de). In addition, transcriptional regulatory elements in non-coding region of the strain were analyzed by the online service system of NSITE (Recognition of Regulatory motifs) of SoftBerry (http://www.softberry.com/berry.phtml).

## Results

### Detection of CIAV Infection in the SPF Layer Breeder Flock

The results of ELISA detection of CIAV antibodies in serum and egg yolk of SPF laying hens were 62% positive and 47% positive, respectively, indicating that the SPF layer breeder flock was infected with the virus (Table 3). CIAV DNA was detected in the pooled organs of embryo samples (35%) by dot blot hybridization assay, while other potential co-infection viruses were negative. In addition, CIAV was detected in 14/40 (35%) embryos by nested PCR and 15/40 (37.5%) embryos by qPCR. All detection methods indicated that embryos from the SPF breeder flock were infected with CIAV.

**Table 3:** Detection of chicken infectious anemia virus by antibody assays and molecular biological methods.

**No. of samples tested for CIAV**			**Antibody assays**			**Molecular biological methods**		
**Tril no**.	**No. of birds**	**No. of embryos**	**No. of seropositive**	**ELISA S/N ratios^**a**^**	**No. positive/total (% positive)**	**No. of embryos positive**			**No. samples positive total^**b**^ (%)**
						**Nucleic acid dot blots**	**Real-time quantitative PCR**	**Nested PCR assay**	
1	1000^c^	/	622	≤ 0.6	62%	NT^f^	NT	NT	NT
2	/	200^d^	94	≤ 0.6	47%	NT	NT	NT	NT
3	**/**	40^e^	NA^f^	NA	NA	14	15	14	35.8%

*^a^ELISA S/N ratios ≤0.6 positive or high protective titers; S/N > 0.6 negative or low titer*.

*^b^The positive rate of the three methods was averaged*.

*^c^Sera samples were obtained from the SPF layer breeder flock used for vaccine production*.

*^d^SPF egg yolk samples were randomly collected*.

*^e^DNA was extracted from pooled organs of chicken embryos*.

*^f^NT: not tested; NA: not applicable*.

### Genome Amplification and Phylogenetic Analysis of CIAV

**Figure 1:**
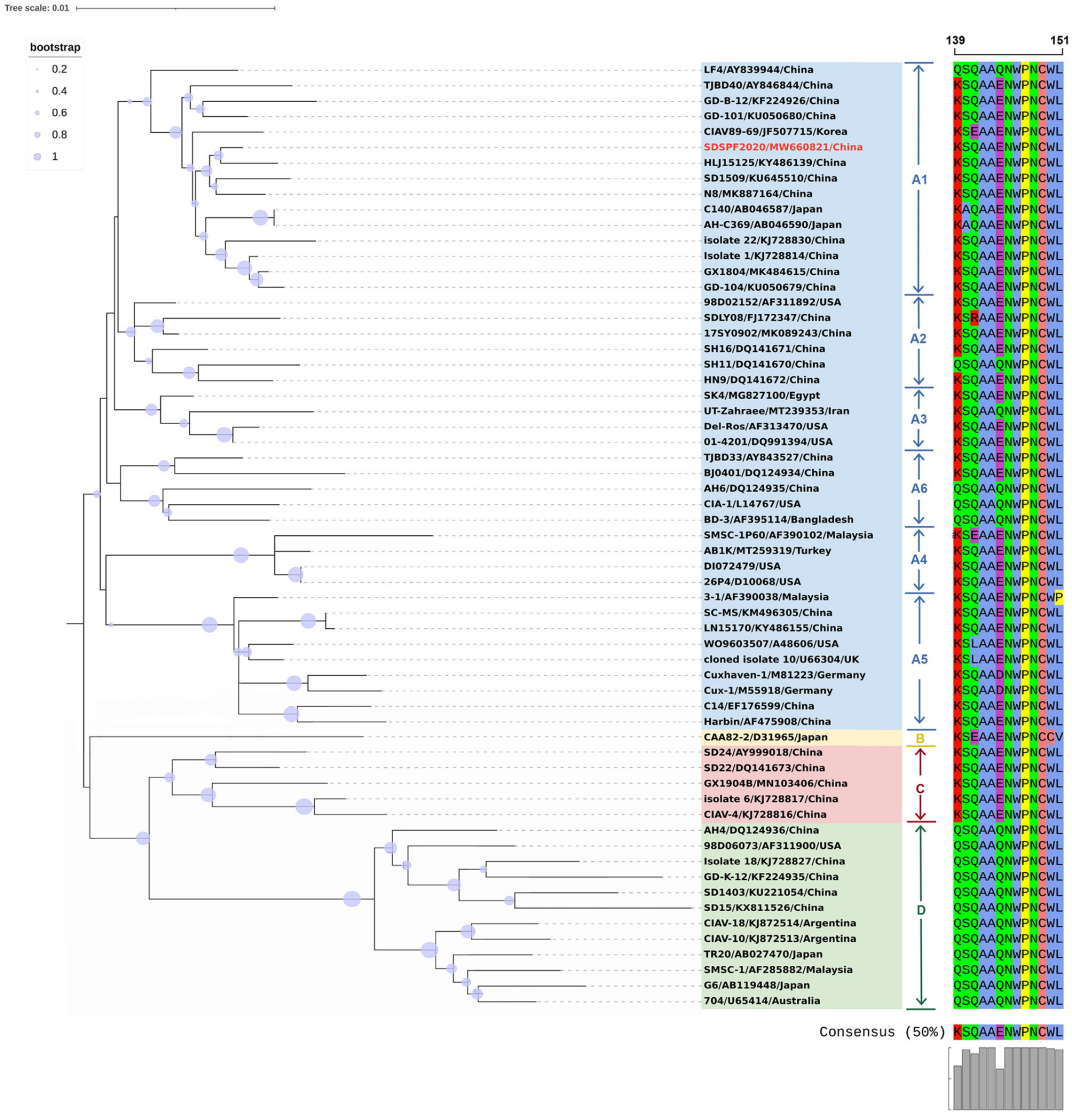
Phylogenetic relationships of SDSPF2020 and other available reference CIAVs in GenBank based on complete genome nucleotide sequences. The phylogenetic trees were generated by neighbor-joining method (1,000 bootstraps) using MEGA X software. The red label represents the strain of the CIAV-SDSPF2020, and a multiple sequence alignment of VP1 protein hypervariable regions (amino acid 139–151) is visualized directly next to the tree. The viruses were clearly divided into 4 genogroups from A to D, and group A included six subgroups.

The full-length genome sequences of CIAV were obtained from the NDV vaccines and SPF chicken embryos by assembling three overlapping fragments. Both genomes consisted of 2,298 nucleotides (nt) with 100% homology. Therefore, the CIAV genomes from the NDV vaccines and SPF chicken embryos were confirmed to be the same strain, named as SDSPF2020, and submitted to GenBank with accession NO. MW660821. The obtained sequence had a high level of nucleotide identity with references (95.8–99.6%). The phylogenetic tree showed that CIAV genomes could be divided into four major clusters (A, B, C, and D) based on previously proposed nomenclature (32). CIAV-SDSPF2020 fell within the A1 group and had the maximal nucleotide sequence identity (99.6%) with the Chinese wild strain HLJ15125 (KY486139), belonging to the same clade (Figure 1). And this new strain was also found to be related to SD1509 and N8 with 99.5% sequence identity, clustered in the same farther branch. These data indicated that the SDSPF2020 strain possessed the typical Asian virus characteristics in the whole genome sequence.

### Protein-Coding Sequence Analysis of SDSPF2020

**Figure 2:**
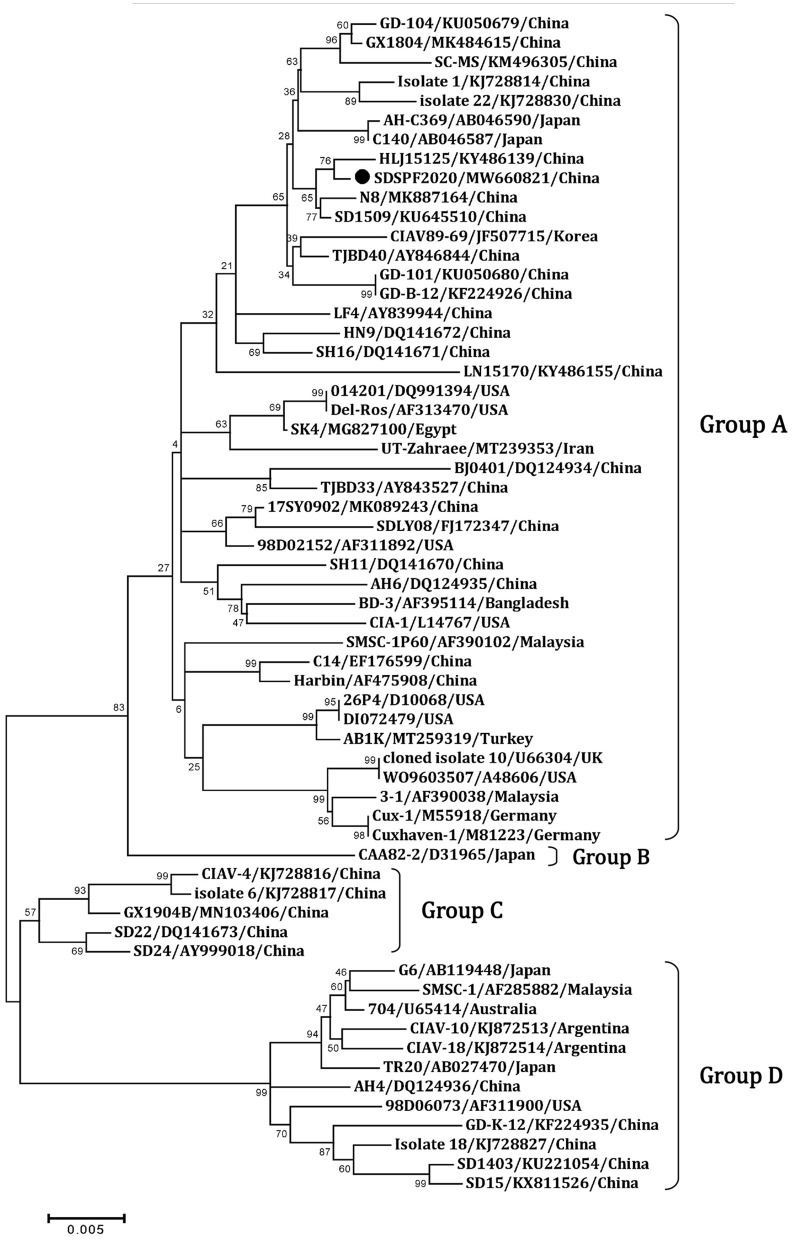
Phylogenetic diagram of viral protein 1 genes among CIAV strains. The CIAV strain determined in this work is highlighted in a black dot, and reference sequences from GenBank were given the name followed by accession number and country. The numbers near the branches indicate bootstrap values. The four major groups were identified as A, B, C and D.

**Figure 3:**
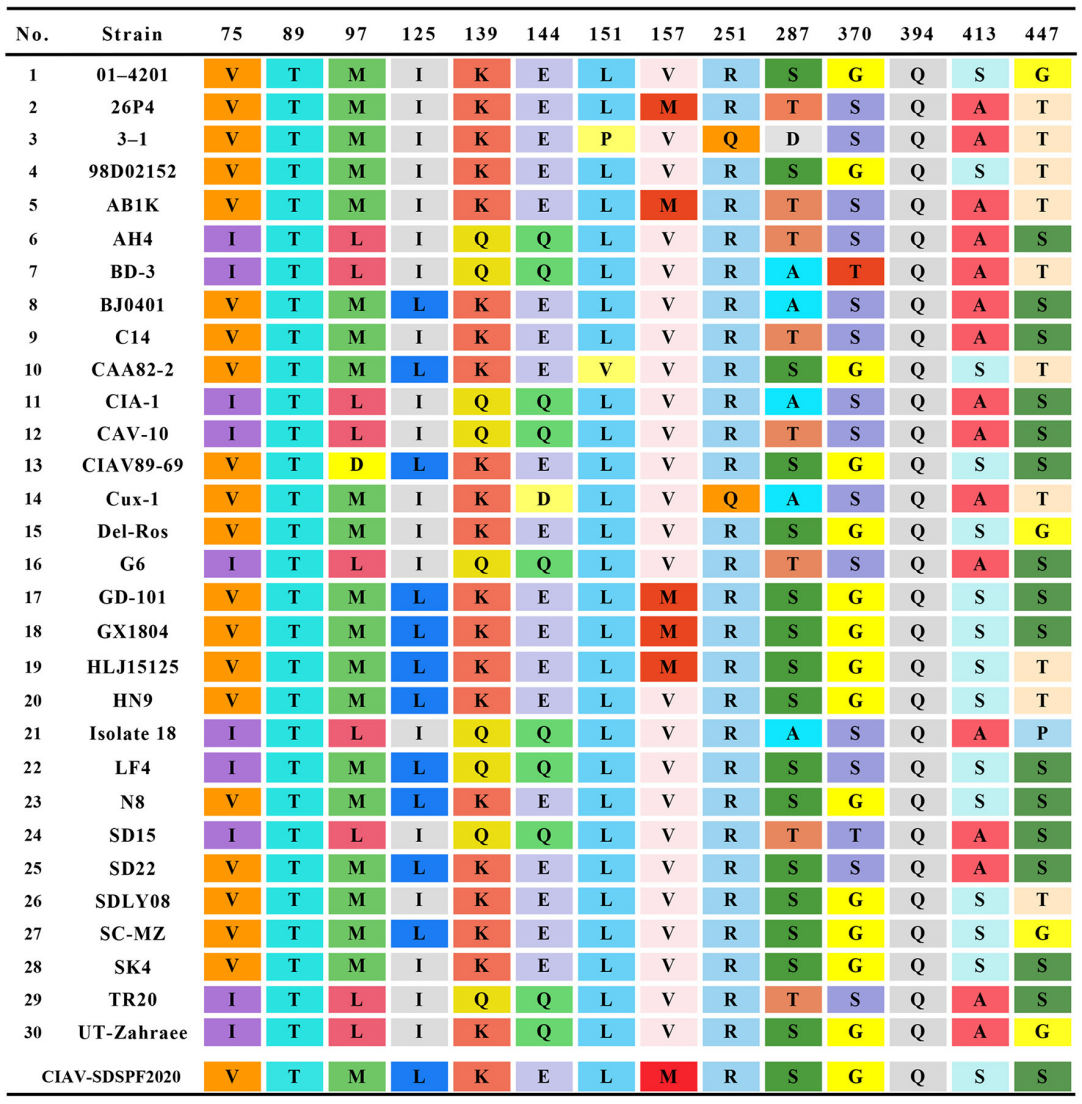
Amino acids at sites of common substitutions in VP1 protein coding sequences of different CIAVs. Each site differences are indicated by different color base box. The last row in the table shows the sites of the new strain.

Similar result was observed in phylogenetic analysis based on VP1 nucleotide sequences. All sequences fell into four gene clusters (Figure 2). The nucleotide homology and amino acid homology of VP1 were 94.4–99.6% and 97.1–100% compared with other reference strains. There are a few differences in nucleotide sequence encoding the VP1 HV region (AAs 139-151) of SDSPF2020, but no unique amino acids were found between SDSPF2020 and the reference sequences (Figure 1). The amino acid sites of VP1 protein manifested the presence of lysine (K) at site 139 and glutamic acid (E) at site 144 in SDSPF2020 strain, and the amino acid sites 125 (L) and 157 (M) were different from most reference CIAV strains (Figure 3). In addition, compared with HLJ15125, synonymous nucleotide substitutions occurred at codons 151 (L), 309 (T), 383 (L), and 420 (Q) in VP1, but codon 447 has a non-synonymous nucleotide difference. However, nucleotide sequences of VP2 and VP3 of SDSPF2020 were relatively conserved. Sequence analysis showed that nucleotide homology of VP2 gene was 99.1–99.8% and amino acid homology was 97.7–100%, while nucleotide identity and amino acid homology in the apoptin gene were 98.9–100% and 96.7–100%, respectively. In addition, no deletion or insertion was found in VP2 and VP3 protein. However, the nucleotides at site 347 and 352 of the VP3 coding sequences were different from the classical reference strain Cux-1, both of which resulted in amino acid differences in the NLS2 domain of VP3 protein.

### Molecular Characterization of Non-coding Region of SDSPF2020

**Figure 4:**
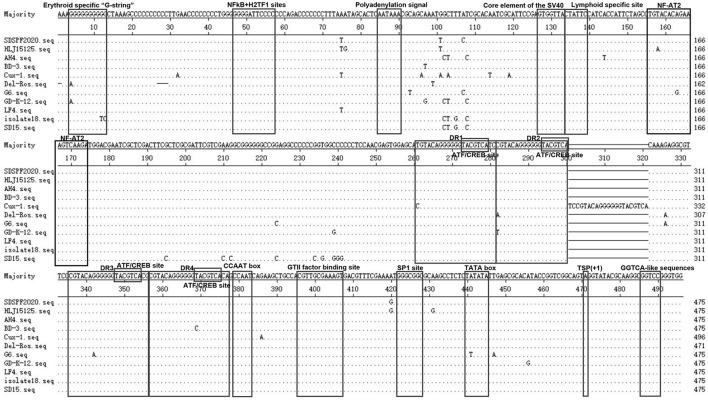
Sequence alignment of the non-coding region sequences of SDSPF2020 and 10 reference strains. The sequences in black frames are the motifs of transcriptional regulatory elements in this study. Nucleotides matching the consensus are indicated with dots and nucleotide differences are indicated with letters.

The Clustal W method was used to analyze the homology of non-coding region fragments of 10 CIAV reference strains. Like the reference strains, the non-coding sequences of SDSPF2020 contained a conservative region of DNA with high G+C content (nucleotide homology 97.0–99.4%). Most motifs in untranslated region of SDSPF2020 were the same as those of the reference strains, while several obvious differences in individual nucleotides existed in SDSPF2020. Transcription factor binding site analysis by NSITE demonstrated that a tandem array of four DR regions was found 4nt upstream of the “CCAAT” box in the non-coding region of SDSPF2020 and most of the reference strains, except for Cux-1, which contains an additional DR copy (Figure 4). The ATF/CREB binding sites (“ACGTCA” consensus sequence) in the DR resembles imperfect hormone response element half-sites (AGGTCA), especially the SP1 recognition site located in close proximity. In addition, it was found that a cluster of GGTCA-like sequences was found downstream of the transcription start point (TSP). In addition, several transcription factor binding sites were all conserved in the non-coding region of SDSPF2020, like GTII factor binding site, TATA box and lymphoid specific transcription factor binding sites.

## Discussion

Since the first strain was isolated from chickens with a vaccine accident in 1979, CIAV has attracted worldwide attention in the poultry industry (42, 43). Although exogenous contamination in vaccines or even SPF chickens has been reported, our results provided evidence for the vertical transmission of CIAV. Recently, we discovered exogenous contamination of CIAV during routine vaccine screening, and confirmed that the contamination also existed in a SPF layer breeder flock and embryos. Next, we analyzed the genetic variation of CIAV-SDSPF2020 strain and tracked its possible origin. Shockingly, the new CIAV genome in NDV vaccines and SPF embryos is the same strain, and has high homology with the Chinese strain HLJ15125 or other wild strains (up to 99.5% or more). It suggested that widespread and multiple epidemics of wild strains may cause the prevalence of CIAV in SPF chickens. These data provided new and crucial information toward a more comprehensive understanding of the origin of CIAV contamination in vaccines.

In this study, we predicted complete amino acid sequences of the new strain and aligned with other CIAV isolates from around the world. The limited genetic variation of CIAV genome is generally <5%, most of which comes from VP1 coding region, because of the extreme conservation of VP2 and VP3 (44, 45). VP1 is the viral capsid and the virulence and pathogenicity of CIAV are associated with critical amino acid residues, such as codon sites 394, 139, and 144 (46, 47). SDSPF2020 strain presented lysine (K) at amino acid site 139 and glutamic acid (E) at site 144, which were related to the rate of replication in culture (48). At amino acid sites 75, 89, 125, SDSPF2020 strain presented valine (V), threonine (T), and leucine (L), respectively, sharing some VP1 amino acids with strains with lower virulent characteristics as previously speculated (32, 47, 49, 50). Therefore, we speculated that SDSPF2020 might be an attenuated virulent strain. In addition, clarifying the properties of transcription regulatory elements provide a better understanding on the mechanisms of host transcriptional regulation and viral pathogenesis. SP1 recognition sites and other transcription factor binding sites are involved in regulation of the CIAV-promoter/enhancer activity (1, 27, 29). Persengiev and Green. reported that the ATF/CREB family of transcriptional regulators have diversified functions in controlling cell proliferation and apoptosis (51). We observed the motifs of these binding sites were also retained in the SDSPF2020 genome. It suggested that those conserved transcriptional regulatory elements may be involved in pathogenicity and viral cytotropism, but the specific regulatory mechanism needs to be further investigated.

CIAV was widely spread in chicken flocks through vertical and horizontal transmission, and the potential route of contamination of vaccines should not be ignored. Numerous studies have proved that exogenous virus contamination existed in live poultry vaccines even under strict surveillance, for example, avian leukosis virus (ALV), Reticuloendotheliosis virus (REV) and fowl adenovirus (FAdV) (52–56). Live attenuated vaccines contaminated with CIAV even in relatively low dose can lead to synergetic pathogenicity and reduce the protective effects against other pathogens (57, 58). By reason of high resistance to common disinfectants and heat treatment, chicken of all ages faces the risk of CIAV infection (17, 59). Epidemiological surveys showed that the positive rate of CIAV tended to be increasing in commercial chicken flocks in recent years, even as high as 87% in some unimmunized chickens in live bird markets (60, 61), which causes substantial economic losses to poultry farming.

However, the fact that the SPF chicken flocks used to produce chicken embryos were infected appears to be treated as if it was relatively rare. Some research groups with SPF flocks throughout the world had encountered these problems in the early 2000 s (15, 62). The work by the Cornell University team demonstrated that CIAV persists in reproductive tissues of sexually mature hens and roosters, and was transmitted vertically to embryos. Brentano et al. also confirmed that CIAV genome exists in the gonads for a long time even in the presence of high neutralizing antibody titers (63). In our experiment, serum collected from a SPF layer breeder flock reacted in ELISA, and viral nucleic acid also existed in organ tissues of chicken embryos. These results demonstrated that SPF layer breeder flocks were widely infected with CIAV and transmitted the virus to chicken embryos through vertical transmission, even under rigid hygiene control and supervision, which may affect the quality of downstream biological products. Nevertheless, the elimination of the virus from infected SPF flocks is a challenging and time-consuming strategy. It is worth noting that the detection of CIAV in embryos and eggshell membranes is an excellent procedure for monitoring potential infections (64). For some vertically transmitted viruses, such as ALV and CIAV, it seems particularly important to establish intensive monitoring and careful management at the parent and grandparent levels of SPF flocks, especially on the hatchery level, which is of great significance to ensure the purity of SPF chicken embryos and vaccines. Thus, it is necessary to continue immunoprophylaxis efforts combined with optimized biosafety programs for the vaccine production.

In conclusion, this study analyzed the whole-genome sequence of CIAV (SDSPF2020) identified from contaminated NDV vaccines, which is consistent with the SPF layer breeder flock, and suggested that it is linked with epidemic wild strains. These findings showed an integrated evidence chain of a vertical transmission route from SPF chickens to vaccines, reminding us that the surveillance of vertically transmitted viruses and biosecurity control of SPF chicken farms should be strengthened continuously to reduce the potential risks of exogenous virus invasion.

## Data Availability Statement

The datasets presented in this study can be found in online repositories. The names of the repository/repositories and accession number(s) can be found here: https://www.ncbi.nlm.nih.gov/genbank/, MW660821.

## Ethics Statement

The animal study was reviewed and approved by Shandong Agricultural University Animal Care and Use Committee and Shandong Agricultural University.

## Author Contributions

YL, JW, LC, and MZ performed the experiments. QW, HZ, and ZC analyzed the data. YL drafted the manuscript. PZ, YW, and SC revised the manuscript. All authors read and approved the final manuscript.

## Funding

This study was funded by the National Key Research and Development Program of China (grant numbers 2018YFD0500106).

## Conflict of Interest

The authors declare that the research was conducted in the absence of any commercial or financial relationships that could be construed as a potential conflict of interest. The handling editor AQ declared a past collaboration with the author PZ.

## Publisher's Note

All claims expressed in this article are solely those of the authors and do not necessarily represent those of their affiliated organizations, or those of the publisher, the editors and the reviewers. Any product that may be evaluated in this article, or claim that may be made by its manufacturer, is not guaranteed or endorsed by the publisher.
